# Essentials of Refraction and the Diseases of the Eye

**Published:** 1890-10

**Authors:** 


ESSENTIALS OF REFRACTION AND THE DISEASES OF THE EYE. By EDWARD JACKSON, A. M., M. D., Professor of Diseases of the Eye in the Philadelphia Polyclinic and College for Graduates in Medicine, etc. Essentials of Diseases of the Nose and Throat. By E. BALDWIN GLEASON, S. B., M. D., Surgeon in Charge of the Nose, Throat and Ear Department of the Northern Dispensary of Philadelphia, formerly Assistant in the Nose and Throat Dispensary of the Hospital of the University of Pennsylvania, etc. Saunders's Question Compends No. 14, with 118 illustrations, 12mo; pp. viii.—276. Philadelphia: W. B. Saunders. 1890.

We have heretofore expressed a favorable opinion of this series of Question Compends. Upon examination this volume seems up to the standard, and will serve the student well upon the subjects of which it treats. We are at a loss to understand why diseases of the *eye*, nose and throat are associated in one volume. The natural arrangement is to connect diseases of the nose, throat and

*ear* in one specialty. This may be seen not only in individual work, but in that of large associations. In most of the latter there is one section, for instance, upon diseases of the eye and another section upon diseases of the nose, throat and ear. This is as it should be. To place a book in the hands of students that does not express this natural arrangement, we believe to be a mistake. We trust that in future editions the publisher will recognize this suggestion. He will then have a volume that teachers can safely recommend.

